# Alleviation of drought stress by mycorrhizas is related to increased root H_2_O_2_ efflux in trifoliate orange

**DOI:** 10.1038/srep42335

**Published:** 2017-02-08

**Authors:** Yong-Ming Huang, Ying-Ning Zou, Qiang-Sheng Wu

**Affiliations:** 1 College of Horticulture and Gardening, Yangtze University, Jingzhou, Hubei 434025, China; 2 Institute of Root Biology, Yangtze University, Jingzhou, Hubei 434025, China; 3 Institute of Fruit and Tea, Hubei Academy of Agricultural Sciences, Wuhan, Hubei 430064, China; 4 Department of Chemistry, Faculty of Science, University of Hradec Kralove, Hradec Kralove 50003, Czech Republic

## Abstract

The Non-invasive Micro-test Technique (NMT) is used to measure dynamic changes of specific ions/molecules non-invasively, but information about hydrogen peroxide (H_2_O_2_) fluxes in different classes of roots by mycorrhiza is scarce in terms of NMT. Effects of *Funneliformis mosseae* on plant growth, H_2_O_2_, superoxide radical (O_2_^·−^), malondialdehyde (MDA) concentrations, and H_2_O_2_ fluxes in the taproot (TR) and lateral roots (LRs) of trifoliate orange seedlings under well-watered (WW) and drought stress (DS) conditions were studied. DS strongly inhibited mycorrhizal colonization in the TR and LRs, whereas mycorrhizal inoculation significantly promoted plant growth and biomass production. H_2_O_2_, O_2_^·−^, and MDA concentrations in leaves and roots were dramatically lower in mycorrhizal seedlings than in non-mycorrhizal seedlings under DS. Compared with non-mycorrhizal seedlings, mycorrhizal seedlings had relatively higher net root H_2_O_2_ effluxes in the TR and LRs especially under WW, as well as significantly higher total root H_2_O_2_ effluxes in the TR and LRs under WW and DS. Total root H_2_O_2_ effluxes were significantly positively correlated with root colonization but negatively with root H_2_O_2_ and MDA concentrations. It suggested that mycorrhizas induces more H_2_O_2_ effluxes of the TR and LRs, thus, alleviating oxidative damage of DS in the host plant.

Drought stress (DS) is usually regarded as one of the major abiotic factors limiting crop growth and yield, including citrus. DS generally triggers oxidative stress, due to the accumulation of reactive oxygen species (ROS) in plant cells. ROS, the byproduct of aerobic metabolism *in vivo*, mainly includes hydroxyl radical (OH·), superoxide radical (O_2_^·−^), and hydrogen peroxide (H_2_O_2_), and it can potentially damage a variety of biological molecules, such as proteins, lipids, and nucleic acids^1^,^2^. At the same time, plants also develop an elaborate antioxidant protection system to eliminate part of ROS in cells and tissues^3^,^4^.

H_2_O_2_ is considered to be not only a toxic molecule inducing oxidative damage, but also an intermediary molecule participating in a variety of physio-biochemical processes in plants. The intermediary function of H_2_O_2_ is closely related to its ability to travel in cellular membranes and migrate into different compartments^2^,^5^. Moreover, H_2_O_2_ is a reliable indicator for the oxidative burst, because it is more stable than O_2_^·−^ 6. The formation of O_2_^·−^ in mitochondria can be measured according to the production of H_2_O_2_ as determined by H_2_O_2_ effluxes^7^,^8^. H_2_O_2_ effluxes may be an important cellular mechanism against oxidative stress^9^,^10^. H_2_O_2_ is effused from the root surface into the rhizosphere in trifoliate orange^11^.

Arbuscular mycorrhizal fungi (AMF), a kind of beneficial soil microorganism, can create a symbiotic association with plant roots forming arbuscular mycorrhizas (AMs), which play a role in the regulation of plant growth^12^,^13^,^14^. AMF can mitigate the detrimental effect of DS through restraining overproduction of ROS^15^,^16^. Less accumulation of ROS in AMF-inoculated plants is due to the enhancement of antioxidant enzyme (e.g. superoxide dismutase and catalase) activities and/or non-enzymatic antioxidants (e.g. ascorbate and glutathione) concentrations^17^,^18^. In addition, AM structures (e.g. arbuscules and hyphae) are involved in H_2_O_2_ accumulation within the roots of the host plant^19^,^20^. Root H_2_O_2_ effluxes are one of the important ROS-scavenging mechanisms in plants subjected to DS, which were found in citrus plants under DS by using the Non-invasive Micro-test Technique (NMT)^11^. NMT can non-invasively preserve the integrity of the sample and provide dynamic information of specific ions/molecules (including K^+^, Na^+^, Ca^2+^, H^+^, Cl^−^, Mg^2+^, H_2_O_2_, IAA, and glucose) near material surfaces^21^,^22^. However, the response of H_2_O_2_ effluxes in different order roots and their contribution to root ROS removal are not fully known under DS conditions, as well as under mycorrhization.

In this context, the objectives of the present study were to investigate the effects of the AM fungus *Funneliformis mosseae* on plant growth, ROS (H_2_O_2_ and O_2_^·−^) levels, malondialdehyde (MDA) concentration, and H_2_O_2_ fluxes in different classes of roots of trifoliate orange [*Poncirus trifoliata* (L.) Raf.] seedlings under WW and DS conditions, and to highlight the distinct role of AMF-alleviated oxidative damage.

## Results

### Mycorrhizal colonization

**Figure 1:**
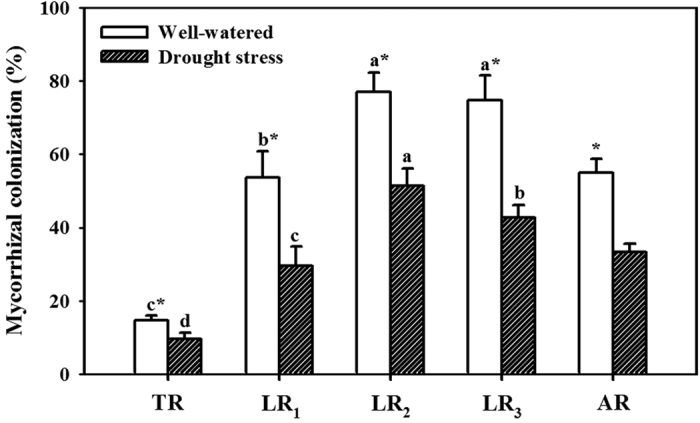
Mycorrhizal colonization in the taproot (TR), first-order lateral root (LR_1_), second-order lateral root (LR_2_), and third-order lateral root (LR_3_) of *Funneliformis mosseae*-colonized trifoliate orange (*Poncirus trifoliata*) seedlings under well-watered (WW) and drought stress (DS) conditions. The average root mycorrhizal colonization (AR) was calculated as (TR+LR_1_+LR_2_+LR_3_)/4. Data (means ± SD, *n* = 4) followed by *different letters* (a, b, c, d) above the bars represent significant differences between the taproot and lateral roots for the same soil water statue, and an *asterisk* (*) above the bars represent significant differences between soil water treatments for the taproot or the same lateral roots at the 5% level.

Root mycorrhizas were found in roots of AMF-inoculated seedlings, but not in roots of non-AMF-inoculated seedlings (Fig. 1). Mycorrhizal seedlings had 54%, 82%, 50%, 74%, and 65% significantly higher AMF colonization in the taproot (TR), first-order lateral root (LR_1_), second-order lateral root (LR_2_), third-order lateral root (LR_3_), and average root (AR) under WW than under DS, respectively. Moreover, AMF mainly colonized LR_2_ and LR_3_ in whole root systems, regardless of WW and DS.

### Plant biomass and RWC

Mycorrhizal seedlings had significantly higher shoot, root, and total biomass than non-mycorrhizal seedlings: 190%, 62%, and 126% under WW and 466%, 148%, and 274% under DS, respectively (Table 1). Compared to non-AMF seedlings, AMF seedlings had 4% and 12% significantly higher RWC under WW and DS conditions, respectively (Table 1).

### Number and surface area of lateral roots

**Figure 2:**
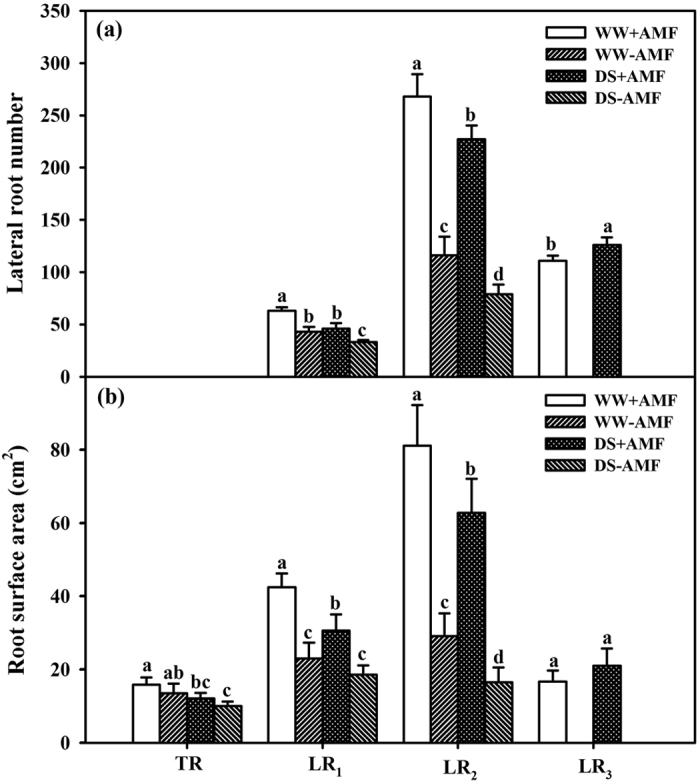
Effect of *Funneliformis mosseae* (AMF) on the number of lateral roots (**a**) and root surface area (**b**) of trifoliate orange (*Poncirus trifoliata*) seedlings under well-watered (WW) and drought stress (DS) conditions. Data (means ± SD, *n* = 4) followed by *different letters* (**a,b,c,d**) above the bars among treatments represent significant differences at the 5% level.

Compared with non-AMF treatment, AMF inoculation significantly increased the number of LR_1_ and LR_2_ by 47% and 131% under WW condition and by 39% and 187% under DS condition, respectively (Fig. 2a). In addition, AMF treatment markedly stimulated LR_3_ formation under WW and DS conditions, but LR_3_ was not found in non-mycorrhizal seedlings. AMF inoculation notably increased the surface area of LR_1_ and LR_2_ by 85% and 182% under WW and by 65% and 280% under DS than non-AMF inoculation, while it did not significantly alter the surface area of the TR (Fig. 2b).

### Concentrations of O_2_^·−^, H_2_O_2_ and MDA

**Figure 3:**
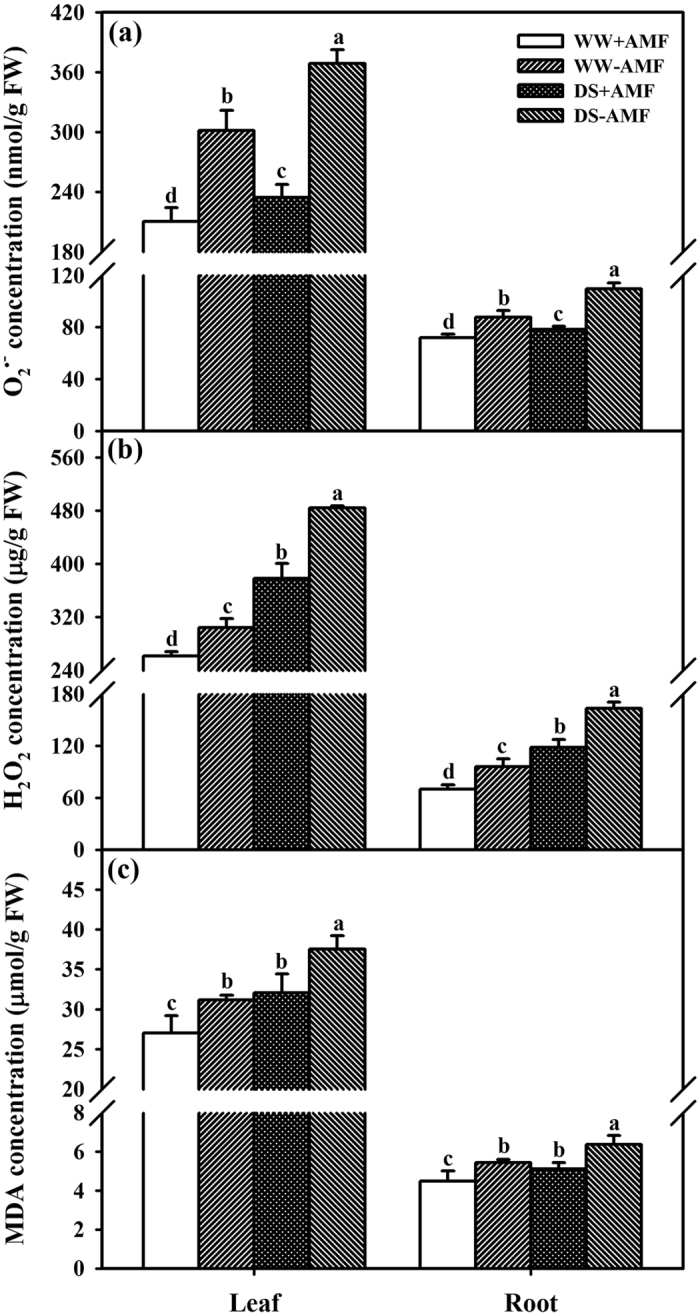
Effects of *Funneliformis mosseae* (AMF) on superoxide radical (O_2_^·−^) (**a**), hydrogen peroxide (H_2_O_2_) (**b**), and malondialdehyde (MDA) (**c**) concentrations of trifoliate orange (*Poncirus trifoliata*) seedlings under well-watered (WW) and drought stress (DS) conditions. Data (means ± SD, *n* = 4) followed by *different letters* (**a,b,c,d**) above the bars among treatments represent significant differences at the 5% level.

AMF seedlings exhibited significantly lower O_2_^·−^ concentrations than non-AMF seedlings regardless of water treatments: 30% and 36% lower in leaves and 18% and 28% lower in roots under WW and DS conditions, respectively (Fig. 3a). Similarly, compared with non-AMF seedlings, AMF seedlings had 14% and 22% significantly lower leaf H_2_O_2_ concentrations and 27% and 28% lower root H_2_O_2_ concentrations under WW and DS conditions, respectively (Fig. 3b). AMF inoculation notably decreased MDA concentrations by 13% and 15% in leaves and 17% and 20% in roots under WW and DS conditions, respectively (Fig. 3c).

### Root H_2_O_2_ effluxes

**Figure 4:**
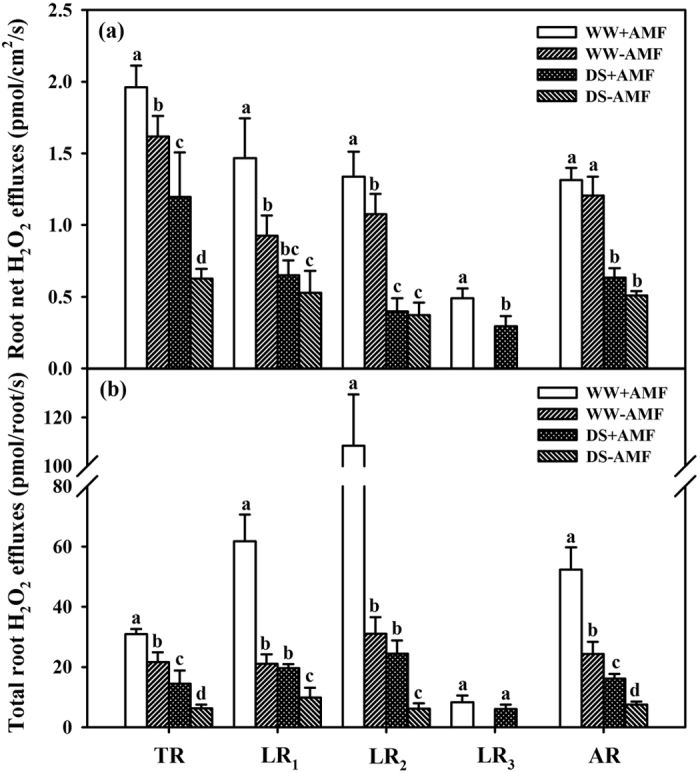
Effects of *Funneliformis mosseae* (AMF) on net root hydrogen peroxide (H_2_O_2_) effluxes (**a**) and total root H_2_O_2_ effluxes (**b**) in the taproot (TR), first-order lateral root (LR_1_), second-order lateral root (LR_2_), and third-order lateral root (LR_3_) of trifoliate orange (*Poncirus trifoliata*) seedlings under well-watered (WW) and drought stress (DS) conditions. Average root H_2_O_2_ effluxes (AR) were calculated as (TR+LR_1_+LR_2_+LR_3_)/4. Total root H_2_O_2_ fluxes of the taproot and lateral roots were calculated as (root surface area × root net H_2_O_2_ effluxes in the corresponding root classes). Data (means ± SD, *n* = 4) followed by different letters (**a,b,c,d**) above the bars represent significant differences between soil water treatments for the taproot or the same lateral roots at the 5% level.

Net root H_2_O_2_ effluxes (real-time effluxes of H_2_O_2_ in the 0.5 cm length from the tip in each order root) were the highest in the TR among different classes of roots, regardless of AMF or soil water status (Fig. 4a). Compared to non-AMF seedlings, AMF seedlings showed significantly higher net root H_2_O_2_ effluxes in the TR, LR_1_, and LR_2_ by 21%, 59%, and 24% under WW condition, respectively. Under DS, AMF inoculation significantly increased net root H_2_O_2_ effluxes by 91% in the TR, but they were not notably altered in LR_1_ and LR_2_, compared with non-AMF inoculation. In addition, net root H_2_O_2_ effluxes in LR_3_ were decreased by 40% in mycorrhizal seedlings under DS conditions than under WW conditions. Average net root H_2_O_2_ effluxes were not significantly different between mycorrhizal and non-mycorrhizal seedlings under WW and DS conditions.

Mycorrhizal seedlings showed 43%, 194%, and 249% higher total root H_2_O_2_ effluxes (real-time effluxes of H_2_O_2_ in whole root systems) in the TR, LR_1_, and LR_2_ under WW and 130%, 99%, and 300% higher under DS condition, respectively, as compared with non-mycorrhizal seedlings (Fig. 4b). The AMF treatment significantly increased average root H_2_O_2_ effluxes by 113% under WW conditions and by 118% under DS conditions, compared to the non-AMF treatment.

### Correlation studies

**Figure 5:**
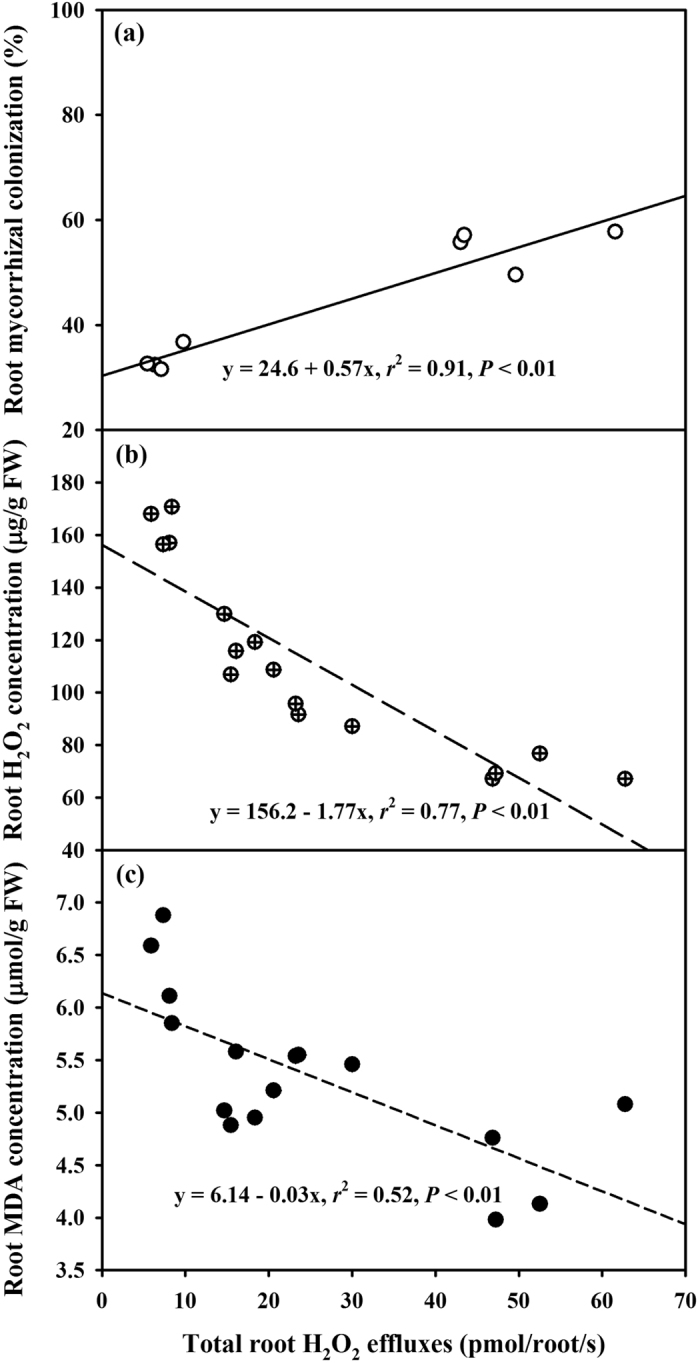
Linear regression between total root hydrogen peroxide (H_2_O_2_) effluxes and root mycorrhizal colonization (**a**, *n* = 8), root H_2_O_2_ concentration (**b**, *n* = 16), or root MDA concentration (**c**, *n* = 16) of trifoliate orange (*Poncirus trifoliata*) seedlings colonized by *Funneliformis mosseae* under well-watered (WW) and drought stress (DS) conditions.

Total root H_2_O_2_ effluxes were significantly positively correlated with root mycorrhizal colonization (Fig. 5a), but negatively correlated with root H_2_O_2_ concentrations (Fig. 5b) and root MDA concentrations (Fig. 5c).

## Discussion

AMF colonization was considerably higher in LRs than in the TR and higher under WW than under DS. This is in agreement with the findings of Ding *et al*.^23^, who reported that *F. mosseae* colonization in LRs of soybean were higher than those in the TR at 56 days after sowing. The decrease of root mycorrhizal colonization under DS compared to under WW ascribed to the reduction of spore germination, root exudates, and root carbohydrate supply^24^. Possibly, greater LR formation in AMF plants, especially LR_2_, could provide more chances to be colonized by mycorrhizal hyphae, resulting in higher mycorrhizal colonization in LRs than in the TR^25^. On the other hand, mycorrhizal plants may tend to have more fine roots and less coarse roots^26^,^27^, which can be successfully colonized.

AMF inoculation with *F. mosseae* alleviated the negative effect of DS on LRs development, indicating the significantly higher number and surface area of LR_1_ and LR_2_ in AMF plants than in non-AMF plants. This is consistent with previous reports^28^,^29^. In general, taproots have a large diameter and play a role in fixing plants and guiding the direction of root growth, while LRs carry out absorption^30^. The LR formation, especially higher order LRs (e.g., 3^rd^ order LR formation in this study), was stimulated by AMF, which helped the host plant to absorb more water and nutrients, thus potentially enhancing drought tolerance in AMF seedlings. Maillet *et al*.^31^ also reported that root branching of the legume *Medicago truncatula* was stimulated by *Rhizoglomus intraradices*. Greater root branching in AMF seedling may be due to the fact that AMF can produce indole-3-acetic acid and polyamines and also further stimulate these phytohormone synthesis in roots^32^,^33^.

Our study showed an efflux behavior of root H_2_O_2_ from roots into the rhizosphere. Compared with WW, DS resulted in lower net root H_2_O_2_ effluxes and total root H_2_O_2_ effluxes, regardless of the TR and LRs. Earlier studies confirmed that H_2_O_2_ effluxes are closely related with cell membrane permeability^34^. As a result, DS strongly destroyed cell membranes and resulted in less effluxes of H_2_O_2_, thereby, inducing the oxidative burst, compared with WW. Root H_2_O_2_ effluxes are a way of physiological metabolism of intracellular H_2_O_2_ overproduction and the response of plant self protection to DS^11^. Bienert *et al*.^2^ first provided molecular genetic evidence that indicated diffusivity of H_2_O_2_ through specific members of the aquaporin family. The decrease in root H_2_O_2_ effluxes by DS would induce more H_2_O_2_ accumulation in roots, thereby resulting in a stronger oxidative burst in plants exposed to DS (e.g., a higher MDA was found under DS than under WW). Generally, net root H_2_O_2_ effluxes gradually decreased with an increase of root order, from the TR to LRs. H_2_O_2_ can be transported long distances across cell membranes from its generation site^35^. Possibly, the taproot as the first arising root in the whole root system has greater capacity to transfer H_2_O_2_ from the extensive original sites (the TR, as well LRs) to the epidermis of the TR, further effluxing into the rhizosphere, as compared with LRs. On the other hand, mycorrhizal structures, including intercellular hyphae and arbuscules, could accumulate H_2_O_2_^19^,^20^, thereby decreasing H_2_O_2_ diffusion in cell membranes. Since root mycorrhizal colonization increased with the increase of root order, from the TP to LRs, a comparatively greater mycorrhizal status in LRs would accumulate more H_2_O_2_ in mycorrhizas, resulting in a decrease of root H_2_O_2_ effluxes, compared with the TR.

AMF colonization by *F. mosseae* significantly increased total root H_2_O_2_ effluxes under WW and DS and net root H_2_O_2_ effluxes in the TR, LR_1_ and LR_2_ under WW and in the TR under DS. Ding *et al*.^23^ reported that H^+^ effluxes in the TR of soybean seedlings were higher than that in LRs, and in extraradical hyphae of the TR than in extraradical hyphae of LRs. Li *et al*.^36^ recently cloned two functional aquaporin genes from *G. intraradices*, which can express in extraradical mycelium under DS conditions. Since H_2_O_2_ diffusion can be through aquaporins^2^, higher root H_2_O_2_ effluxes in AMF seedlings than in non-AMF seedlings might be involved in the H_2_O_2_ release of aquaporins by extraradical hyphae. Total root H_2_O_2_ effluxes were significantly positively correlated with root AMF colonization, strengthening the functioning of AMs on H_2_O_2_ effluxes. Further studies are needed to demonstrate whether AM extraradical hyphae release H_2_O_2_, and if H_2_O_2_ in root cells is transported from arbuscules to extraradical hyphae and then completely effluxed through special aquaporins. In whole root systems, LR_2_ had the highest total root H_2_O_2_ effluxes, which is due to the fact that LR_2_ had a distinctly higher root mycorrhizal colonization, root surface area and number. In the present study, the significantly negative correlation of total root H_2_O_2_ effluxes with root H_2_O_2_ or MDA concentrations indicated that root H_2_O_2_ effluxes result in less accumulation of root H_2_O_2_, thus mitigating damage of membrane lipid peroxidation.

In conclusion, inoculation with *F. mosseae* induced considerably higher root H_2_O_2_ effluxes in LRs and the TP, which might result in a lower oxidative damage in roots under DS.

## Methods

### Experimental design

The experiment was a completely randomized block design with two factors: (1) two mycorrhizal treatments, inoculation with *Funneliformis mosseae* (+AMF) and without *F. mosseae* (−AMF), and (2) two soil water levels, well-watered (WW, 75% of maximum water holding capacity of soil) and drought stress (DS, 55% of maximum water holding capacity of soil). Each of the four treatments had four replicates, with a total of 16 pots.

### Mycorrhizal inoculum

The AM fungus strain, *Funneliformis mosseae* (Nicol. & Gerd.) Schüßler & Walker, was isolated from the rhizosphere of *Incarvillea younghusbandii* in Dangxiong (90°45′E and 29°31′N, 4,300 m above the sea level), Tibet, and further propagated using white clover (*Trifolium repens*) as a host plant for 16 weeks under potted conditions. For the AMF treatment (+AMF), 100 g inoculum (15 spores/g), including spores, infected root segments of white clover plants, and growth substrates was mixed with the 1.2 kg soil at transplanting. The non-AMF-inoculated treatment (-AMF) received the same amount of sterilized inoculums together with 2 mL inoculums filtrate (25 μm filter) to maintain a similar microbial community except for *F. mosseae*.

### Plant material

Seeds of trifoliate orange were surface-sterilized with 70% alcohol for 10 min, rinsed five times with distilled water, and placed in autoclaved (0.11 Mpa, 121 °C, 2 h) sands at 26 °C for germination. Subsequently, four-leaf-old seedlings of the same size were transferred into a 1.1 L plastic pot containing 1.2 kg autoclaved (0.11 Mpa, 121 °C, 2 h) soil from a citrus orchard of the Yangtze University campus. The soil collected here had a pH of 6.0, 12.1 mg/kg KMnO_4_-N, 15.7 mg/kg Bray-P, 22.3 mg/kg neutral NH_4_OAc-K, and 38% maximum water holding capacity. All the seedlings were placed in a glasshouse (photosynthetic photon flux density was 982 μmol m^−2^ s^−1^, day/night temperature 27/20 °C, and relative humidity 80%) from March 24 to August 19, 2014.

### Soil water treatment

According to Zou *et al*.^11^, WW treatment was selected as 75% of maximum water holding capacity, and DS was considered as 55% of maximum water holding capacity of soil. Before DS was begun, soil water of these pots was kept in WW. After 99 days, half of the seedlings were kept in DS status for 50 days and the other seedlings were still kept in WW status for 50 days. The soil water levels in the pots were determined daily through weighing, and the amount of water lost was supplied to each pot to reach the designed soil water levels. During the experiment, a 50 mL of Hoagland solution with half of P strength was biweekly added into each pot for normal growth of seedlings.

### Variable determinations

After 50 days of DS treatment, all the plants were harvested, and plant height, stem diameter and leaf number per plant were recorded. Subsequently, the plants were divided into shoots and roots, whose fresh weights were measured. According to Hackket^37^, root systems were divided into the TP and LRs. The number of LR_1_, LR_2_, and LR_3_ was carefully counted. Ten 1 cm long root segments per pot of the TR, LR_1_, LR_2_, and LR_3_ were stained with 0.05% (w/v) trypan blue for 5 min^38^ and observed by the LEICA DME bio-microscope for AM structures. Root AMF colonization was calculated as the percentage of colonized root lengths by AMF against observed total root length.

Different classes of roots per plant were scanned with an Epson Perfection V700 Photo (Seiko Epson Corp, Japan), and then the scanned root images were analyzed to obtain the root surface area using a WinRHIZO professional 2007b (Regent Instruments Incorporated, Canada).

The fourth fully expanded top leaf was used to determine leaf RWC by the method outlined by Bajji *et al*.^39^. The leaf RWC was calculated by the following formula: RWC (%) = 100 × (FW-DW)/(SW-DW), where FW refers to fresh weight, DW to dry weight (oven-dried, at 75 °C for 48 h), and SW to saturated weight after leaf rehydration for 24 h.

MDA, a by-product of oxidative damage to lipids in tissues, was measured following the method described by Sudhakar *et al*.^40^. Fresh samples (0.1 g) of leaves and roots were homogenized with 5 mL 0.1% (w/v) trichloroacetic acid and centrifuged at 3,000 × *g* for 10 min. The mixture including 0.5 mL supernatant and 1.5 mL 0.67% thiobarbituric acid was incubated at 100 °C for 0.5 h and centrifuged at 3,000 × *g* for 10 min. The absorbance at 450, 532, and 600 nm was recorded, and the MDA concentration (μmol/L) was calculated by the formula: 6.45 (A_532_ − A_600_) − 0.56A_450_.

The H_2_O_2_ concentration in leaves and roots was determined according to Velikova *et al*.^41^. Fresh samples (0.1 g) were homogenized with 5 mL 0.1% (w/v) trichloroacetic acid and centrifuged at 12,000 × g for 15 min. A 4 mL mixture was comprised of 1 mL 10 mM potassium phosphate buffers (pH 7.0), 2 mL 1 M KI, and 1 mL supernatant. The absorbance at 390 nm was recorded.

The levels of O_2_^·−^ in tissues were measured using the protocol described by Wang and Luo^42^. Fresh leaf or root samples (0.15 g) were homogenized in 5 mL 0.1 M phosphate buffer (pH 7.8) and centrifuged at 4,000 × g for 10 min at 4 °C. The 0.5 mL supernatant was mixed with 0.5 mL 50 mM phosphate buffers (pH 7.8) and 0.1 mL 10 mM hydroxylamine chloride for reaction for 1 h at 25 °C. Subsequently, 1 mL 17 mM sulfanilamide and 1 mL 7 mM α-naphthylamine were added to the mixture at 25 °C for 20 min, and the absorbance was recorded at 530 nm.

Net root H_2_O_2_ fluxes in a 0.5 cm length from the tip of the taproot and different order lateral roots were measured using the Scanning Ion-Selective Electrode Technique (SIET) system (BIO-001A, Younger USA Sci. & Tech. Corp., Amherst, MA, USA) in the Xuyue Beijing NMT Service Center (Xuyue Beijing Sci. and Tech. Co., Ltd., Beijing, China). An H_2_O_2_-sensitive microsensor was polarized at +600 mV against an Ag/AgCl reference electrode before use^6^. Root systems *in vivo* were placed in a Petri dish and immersed in an assay solution, comprised of 0.1 mM CaCl_2_, 0.1 mM KCl, and 0.3 mM MES (pH 6.0). The H_2_O_2_ microsensor was placed 30 μm from the root surface. Net root H_2_O_2_ fluxes were calculated by Fick’s law of diffusion: *J* (pmol/cm^2^/s) = −*Do* × (*dc*/*dx*), where *Do* indicates the diffusion constant, *dc* the concentration gradient, and *dx* the distance (30 μm)^43^. Total root H_2_O_2_ fluxes were counted according to *J* × surface area of the lateral root.

### Statistical analysis

Data (means ± SD, *n* = 4) were statistically analyzed by the variance (ANOVA) with SAS 8.1 software (SAS Institute Inc., Cary, NC, USA), and the significant differences between the treatments were tested by Duncan’s Multiple Range Tests at *P* < 0.05.

## Additional Information

**How to cite this article**: Huang, Y.-M. *et al*. Alleviation of drought stress by mycorrhizas is related to increased root H_2_O_2_ efflux in trifoliate orange. *Sci. Rep.* **7**, 42335; doi: 10.1038/srep42335 (2017).

**Publisher's note:** Springer Nature remains neutral with regard to jurisdictional claims in published maps and institutional affiliations.

## Figures and Tables

**Table 1:** Effects of *Funneliformis mosseae* (AMF) on plant biomass and leaf relative water content of trifoliate orange (*Poncirus trifoliata*) seedlings under well-watered (WW) and drought stress (DS) conditions.

Treatments	Biomass (g FW/plant)			Leaf relative water content (%)
	Shoot	Root	Total	
WW+AMF	4.17 ± 0.31a	2.33 ± 0.06a	6.50 ± 0.29a	89.40 ± 1.01a
WW−AMF	1.44 ± 0.58c	1.44 ± 0.27c	2.88 ± 0.85c	86.34 ± 0.87b
DS+AMF	3.00 ± 0.23b	1.98 ± 0.10b	4.98 ± 0.28b	86.60 ± 1.87b
DS−AMF	0.53 ± 0.08d	0.80 ± 0.20d	1.33 ± 0.27d	76.93 ± 2.95c

Note: Data (means ± SD, *n* = 4) followed by different letters among treatments represent significant differences at the 5% level.
